# The Association between Food Addiction and Early Maladaptive Schemas in Overweight and Obese Women: A Preliminary Investigation

**DOI:** 10.3390/nu9111259

**Published:** 2017-11-17

**Authors:** Claudio Imperatori, Marco Innamorati, David Lester, Massimo Continisio, Michela Balsamo, Aristide Saggino, Mariantonietta Fabbricatore

**Affiliations:** 1 Department of Human Science, European University of Rome, Via degli Aldobrandeschi 190, 00163 Roma, Italy; Marco.Innamorati@unier.it (M.I.); m.continisio@alice.it (M.C.); mfabbricatore@tiscali.it (M.F.); 2 Stockton University, 101 Vera King Farris Drive, Galloway, NJ 08205-9441, USA; David.Lester@stockton.edu; 3 Department of Pychological, Health and Territorial Sciences, University of Chieti-Pescara, Via dei Vestini 31, 0871, 3551Pescara, Italy; michela.balsamo@libero.it (M.B.); aristide.saggino@unich.it (A.S.)

**Keywords:** food addiction, early maladaptive schemas, binge eating severity, obesity, overweight

## Abstract

In recent years, there has been a growing focus on early maladaptive schemas (EMSs) as core features associated with eating psychopathology. The aims of the present study were to assess in overweight and obese women: (i) the association between dysfunctional eating patterns (i.e., food addiction and binge eating) and EMSs, and (ii) the association between food addiction and EMSs after controlling for potential confounding variables (i.e., binge eating severity and psychopathology). Participants were 70 overweight and obese women seeking low-energy-diet therapy. The patients were administered self-report measures investigating food addiction, binge eating, EMSs, anxiety symptoms, and depressive symptoms. Food addiction severity was strongly associated with all main schema domains. Binge eating severity was positively related to disconnection/rejection (*r* = 0.41; *p* < 0.01), impaired limits (*r* = 0.26; *p* < 0.05), and other-directedness domains (*r* = 0.27; *p* < 0.05). The disconnection/rejection schema was independently associated with food addiction severity, after controlling for the presence of other potential confounding variables (i.e., binge eating severity and psychopathology) suggesting that this domain may be a crucial factor for the development of food addiction.

## 1. Introduction

Obesity (i.e., body mass index (BMI) ≥ 30 kg/m^2^) and being overweight (BMI ≥ 25 kg/m^2^) are widespread medical conditions, caused by multiple and different factors (e.g., genetic and psychosocial variables) [1], and they have recently become a serious problem in developed countries [2]. Although not recognized as eating disorders (EDs) by the Diagnostic and Statistical Manual of Mental Disorders Fifth Edition (DSM-5) [3], obesity and being overweight are often related to psychiatric disorders [4] and several dysfunctional eating patterns, such as binge eating [5], night eating [5], and grazing behavior (i.e., the consumption of smaller amounts of food over extended periods of time) [6].

In the last few years, the construct of food addiction (FA) has also been described as a disordered eating behavior frequently observed in both obese and overweight patients, with a prevalence of roughly 25% (for a review and meta-analysis on FA prevalence, see [7]). FA has been conceptualized as a chronic and relapsing condition characterized by intense cravings for hyper-palatable foods as well as other typical symptoms of substance-related and addictive disorders (e.g., tolerance and withdrawal symptoms) [8]. Although this concept was originally introduced to better understand abnormal eating patterns in obese and overweight patients [9,10], FA is frequently diagnosed in healthy-weight individuals (about 11%) and in patients with EDs (about 58%) [7]. For example, the prevalence of FA in individuals with binge eating disorder (BED) ranges between 33.3% and 61.7% across published studies [11,12,13,14], suggesting that, although FA and BED are strongly related, they do not completely overlap [15]. Therefore, it has been recently hypothesized that FA may exist as a trans-nosographic construct in obese and overweight patients as well as in all ED patients, especially in those with BED and bulimia nervosa [16,17].

According to cognitive behavioral models, maladaptive thoughts, and dysfunctional beliefs about shape, weight, and eating concerns are crucial factors in developing and maintaining EDs [18,19] and obesity [20,21]. In recent years, there has been a growing focus on early maladaptive schemas (EMSs) as a core feature associated with eating psychopathology [22]. Within the “Schema Therapy” theoretical framework [23,24], EMSs are defined as “*extremely stable and enduring themes, comprised of memories, emotions, cognitions, and bodily sensations regarding oneself and one’s relationship with others, that develop during childhood and are elaborated on throughout the individual’s lifetime, and that are dysfunctional to a significant degree*” [24]. EMSs are considered to have developed early in childhood through the interaction between child temperament and the early experiences of deprivation and/or frustration [25].

Young et al. [24] proposed 18 EMSs clustered into five main domains: (i) *disconnection and rejection* (i.e., the expectation that one's need for security, safety, and acceptance will not be met in a predictable manner), (ii) *impaired autonomy and performance* (i.e., expectations about oneself and the environment that interfere with one's ability to separate, survive, or perform successfully), (iii) *impaired limits* (i.e., a deficiency in internal limits, responsibility to others, or long-term goal orientation), (iv) *other directedness* (i.e., an excessive focus on the desires, feelings, and responses of others, at the expense of one’s own needs, in order to gain love, maintain connection, or avoid retaliation), and (v) *over-vigilance and inhibition* (i.e., an excessive emphasis on suppressing spontaneous feelings, impulses, and choices, or meeting rigid, internalized rules and expectations about performance).

A recent review, of 32 studies exploring interactions between eating psychopathology and EMSs, showed that EDs are characterized by severe EMSs, especially the disconnection/rejection and impaired autonomy/performance domains [22]. Specifically, Pugh [22] reported that (i) EMSs were more pronounced in patients with EDs compared to both healthy controls and clinical groups (e.g., patients with alcohol and substance use disorder); (ii) binge eating behaviors were positively associated with several EMSs (emotional inhibition appears to be the most robust predictor of binge frequency); (iii) defectiveness/shame and emotional deprivation EMSs were the most reliable predictor of purging behaviors; (iv) defectiveness/shame and failure to achieve EMSs appear to be the most robust predictors of restrictive attitudes; and (v) body dissatisfaction was positively related to all five schema domains.

Although several studies [26,27] have reported an association between FA and adverse childhood experiences (e.g., sexual abuse and emotional and physical neglect), to the best of our knowledge, no studies have directly investigated the relationship between FA and EMSs. Furthermore, few reports have explored EMSs in overweight and obese patients. For example, Anderson et al. [28] showed that, compared to normal-weight adults, obese adults were reported with a greater severity of maladaptive schemata (i.e., social isolation, defectiveness/shame, and failure to achieve), even after controlling for demographic variables and BED diagnosis. Moreover, in the obese group, the severity of maladaptive schema scores was positively related to both mood disturbances and dysfunctional eating tendencies, while no significant association was detected between EMSs and BMI [28]. Poursharifi et al. [29] reported that some aspects of identity (e.g., social and relational identities) were positively associated with EMSs in obese patients. More recently, da Luz et al. [30] observed that, compared to normal-weight individuals, patients with morbid obesity (BMI > 40 kg/m^2^) had higher scores for the insufficient self-control/self-discipline schema, even though this difference was not significant when mental health status was controlled for. Finally, it has been reported that, compared to normal-weight adolescents, overweight [31] and obese [32] adolescents reported higher EMSs.

Therefore, on the basis of the above analysis of the literature, the aims of the present study were to assess, in overweight and obese women, (i) the association between dysfunctional eating patterns (i.e., FA and binge eating) and EMSs, and (ii) the association between FA and EMSs after controlling for potential confounding variables (e.g., binge eating severity and psychopathology), which are known to be strongly related to FA [33].

## 2. Material and Methods

### 2.1. Participants

Participants were 70 overweight and obese women consecutively admitted between December 2016 and July 2017 to a medical center in Rome (Italy), specializing in the nutritional treatment of obesity using low-energy-diet therapy. Patients had an average BMI of 29.65 kg/m^2^ (SD = 4.25: range: 25.04–42.31) and an average age of 46.09 years (SD = 9.95: range: 18–71). Patients were assessed at entry into the study. Inclusion criteria were an age of 18 or higher and a BMI of 25 kg/m^2^ or higher. Exclusion criteria were a history of neurologic or psychiatric diseases, purging and non-purging compensatory behaviors, and the presence of any condition affecting the ability to complete the assessment, as well as denial of informed consent. A checklist with dichotomous items was used to assess the inclusion criteria and exclusion criteria. The clinical and socio-demographic characteristics of the sample are listed in Table 1. After receiving information about the aims of the study, all patients provided written consent for participation. The study was in accordance with the Helsinki declaration standards and was approved by the ethics review board of the European University of Rome (Prot. N.005/16).

### 2.2. Measures

All of the participants were administered the Italian version of the Yale Food Addiction Scale (YFAS) [34], the Young Schema Questionnaire Long Form, Third Edition (YSQ-L3) [35], the Binge Eating Scale (BES) [36], and the Hospital Anxiety and Depression Scale (HADS) [37]. 

The YFAS [38] is a 25-item self-report measure of addictive eating behaviors with regard to high fat/sugar foods. The YFAS includes dichotomous and Likert-type scale formats with two scoring alternatives: a symptom count version and a diagnostic version. It is based on the 4th edition of Diagnostic and Statistical Manual of Mental Disorders (text revision) (DSM-IV-TR) [39] criteria for drug addiction. These criteria include (i) a substance taken in a larger amount and for a longer period than intended; (ii) a persistent desire or repeated unsuccessful attempts to quit the use of the substance; (iii) a large amount of time/activity necessary to obtain, use, or recover from the use of the substance; (iv) important social, occupational or recreational activities discontinued or reduced because of the use of the substance; (v) continuing use of the substance despite knowledge of the adverse consequences associated with its use; (vi) tolerance; and (vii) withdrawal symptoms. A categorical cut off point is met when three symptoms and clinically significant impairment or distress from eating are present. The YFAS has demonstrated satisfactory psychometric properties in different samples and countries [16]. In the present study, Cronbach’s α for the YFAS was 0.85.

The YSQ-L3 [40] is a 232-item questionnaire assessing 18 EMSs, according to the principles of Schema Therapy [24], grouped into five domains: (i) Disconnection/Rejection; (ii) Impaired Autonomy/Performance; (iii) Impaired Limits; (iv) Other-Directedness; and (v) Over-Vigilance/Inhibition. The items are rated on a 6-point Likert-type scale ranging from 1 (“*it is completely untrue for me*”) to 6 (“*it describes me perfectly*”). Higher scores reflect the greater presence of maladaptive schemas. Several studies have documented satisfactory psychometric properties for this scale [24]. In the current study, Cronbach’s α for the whole scale was 0.98, and ranged between 0.90 for Impaired Limits and 0.97 for Over-Vigilance/Inhibition.

The BES [41] is a 16-item self-report scale assessing binge eating severity as well as behavioral manifestations and the feelings/cognitions manifestations related to such behavior. When rating each item, the respondent has to choose between 3 and 4 response statements of increasing severity for each question. The total score ranges from 0 to 46. According to Ricca et al. [42], a cutoff score of 17 discriminates between patients with and without a clinical level of binge eating. Cronbach’s α for the present sample was 0.88.

The HADS [43] is a 14-item questionnaire assessing anxiety and depression symptoms. The items are rated on a 4-point Likert-type scale (from 0 to 3). Total scores range from 0 to 21 for each subscale, with greater scores reflecting a greater presence of anxiety and depression symptoms. Although it was originally developed to screen for depression and anxiety in a hospital setting, the HADS is also used to assess anxiety and depression symptoms in the general population [43]. Cronbach’s α for the present sample was 0.83 and 0.84, respectively, for the anxiety and depression subscales.

### 2.3. Statistical Analyses

All analyses were performed with the SPSS 19.0 statistical package for the social sciences (IBM, Armonk, NY, USA). The relationships between the variables were assessed using Pearson’s *r* correlation coefficients.

All the variables significantly associated with FA at the bivariate level were inserted in a hierarchical regression analysis with YFAS total score (i.e., symptom count) as the dependent variable. We used the standard method of entry (also known as the “enter method”), whereby at each step all independent variables are entered into the equation simultaneously. The associations were reported as standardized beta coefficients and their *p* values.

## 3. Results

In the sample, there were 16 patients (22.9%) who met the criteria for a diagnosis of FA according the YFAS, 20 (28.5%) who met the criteria for clinical-level binge eating (BES > 17), and 12 (17.1%) who met both criteria. Of those who met FA criteria, 75% (*N* = 12) also satisfied criteria for clinical-level binge eating, whereas, of patients with clinical-level binge eating, 60% (*N* = 12) also satisfied the criteria for FA.

Correlations between the variables are reported in Table 2. FA symptoms were strongly associated with binge eating severity (*r* = 0.63; *p* < 0.01), the five dimensions of YSQ-L3 (*r* > 0.39), and with both depressive symptom severity (*r* = 0.43; *p* < 0.01), and anxiety symptom severity (*r* = 0.31; *p* < 0.01). On the YSQ-L3, binge eating severity was positively associated with disconnection/rejection (*r* = 0.41; *p* < 0.01), impaired limits (*r* = 0.26; *p* < 0.05), and other-directedness domains (*r* = 0.27; *p* < 0.05). Binge eating symptoms were also significantly associated with depressive symptoms (*r =* 0.38; *p* < 0.01) and BMI (*r* = 0.28; *p* < 0.05). Anxiety symptoms were positively associated with all five YSQ-L3 domains (*r* = 0.38; *p* < 0.01) with the exception of impaired limits. Depressive symptoms were positively associated with all five domains (*r* = 0.32; *p* < 0.01) Finally, BMI was not related to any EMSs.

Variables significantly associated with FA at the bivariate level were inserted as independent variables into a hierarchical linear regression analysis with YFAS total score (i.e., symptom counts) as the criterion. The models explained between 16.0% and 50.0% of the variability of the data (Table 3). In the last block, when controlling for the presence of other variables, Disconnection/Rejection domain was independently associated with YFAS total score; thus, a more severe Disconnection/Rejection maladaptive schema was associated with more FA symptoms (standardized beta coefficient = 0.29; *p* < 0.05).

## 4. Discussion

The major aim of the present study was to assess the association between FA and EMSs in overweight and obese women, controlling for potential confounding variables (e.g., binge eating severity and psychopathology). Consistent with previous studies assessing the association between EMSs and eating psychopathology [22], our results showed that FA symptoms are positively related to all five schema domains of the YSQ-L3. Our results also showed that binge eating severity was positively associated with three maladaptive schemas (i.e., disconnection/rejection, impaired limits, and other-directedness domains). No significant association was observed between BMI and EMSs scores, suggesting that dysfunctional schemas in overweight and obese women are likely related to their individual mental health (i.e., dysfunctional eating patterns and/or psychopathology) and not to their weight, as has been previously hypothesized for patients with morbid obesity [30]. Finally, our regression analysis showed that the disconnection/rejection schema score was independently associated with FA symptoms, after controlling for the presence of other potential confounding variables (e.g., psychopathology and binge eating severity), suggesting that this domain may be a crucial factor for the development of FA.

Our study differs from, and adds to, previous findings from investigations of the association between EMSs and FA. We used a sample of obese and overweight women and controlled for the presence of potential confounding variables (e.g., psychopathology and binge eating severity). Our results are in line with previous findings showing that EDs are characterized by severe EMSs, especially the disconnection/rejection schema [22]. Our data are also consistent with studies reporting a strong association between the disconnection/rejection schema and substance-related and addictive disorders [44,45,46,47]. For example, it has been reported that EMSs, especially the disconnection/rejection schema, play an important role in the prediction of addiction [44]. Furthermore, it has been observed that the disconnection/rejection schema was the most robust predictor of adult risky sexual behaviors [46] and was positively related to both sexual and substance addiction severity in a sample of 260 patients in residential treatment for substance use disorders [45].

The disconnection/rejection domain is characterized by a fear that one’s basic needs for security, safety, and acceptance will not be met and that significant others will abuse and/or abandon the person [24]. It has been observed that dysfunctional coping responses are often used for controlling EMSs, such as avoidance behavior, which can result in addictive behaviors as a defense mechanism [47,48]. Therefore, the association between FA symptoms and the disconnection/rejection domain may reflect a dysfunctional coping strategy consisting of the use of hyper-palatable foods (i.e., “comfort foods”) to escape from the unpleasant state, which arises from the activation of dysfunctional schema triggered by environmental cues and related to intense emotional experiences first encountered in childhood (e.g., the fear of being abused and/or abandoned by the significant people). This hypothesis is in line with recent studies reporting an association between FA and several adverse childhood experiences such as sexual abuse and emotional and physical neglect [26,27].

Our study may be also useful in the current debate about the relationship FA and BED [15]. Consistently with previous findings [11,12,13,14,15], our results (i.e., the prevalence of FA in patients with a clinical level of binge eating, as well as the strong correlation between YFAS and BES total scores) showed that, although FA and BED are closely related, they do not completely overlap. Burrows and colleagues [15] recently observed that, while FA strongly overlaps with the emotional component of binge eating (e.g., feeling guilty after binge eating), it is less strongly related to the behavioral component of binge eating (e.g., eating quickly). Our data seem to suggest that another difference between FA and BED may be related to the EMSs. Indeed, while FA symptoms were positively related to all five schema domains, binge eating severity was positively associated only with three main maladaptive schemas (i.e., disconnection/rejection, impaired limits, and other-directedness domains), suggesting a greater influence of EMSs on addictive eating behaviors than on binge eating psychopathology. Although this interpretation remains speculative, it might be useful in guiding future research in large sample in order to investigate directly how different types of EMSs may selectively affect various types of dysfunctional eating patterns (i.e., FA, BED, FA, and BED).

Although the present findings are interesting, some issues limiting their generalizability should be considered. First, we have focused only on obese and overweight females with, therefore, a limited BMI range. Secondly, we have used only self-report measures, which may be potentially affected by a social desirability bias [49,50]. Third, we have used the old version of YFAS, which is based on the DSM-IV-TR criteria for substance addiction [39]. Fourth, the cross-sectional nature of the study precludes causal interpretations among the variables. Finally, the small sample size makes it difficult to draw definitive conclusions from our data, which must be considered only as preliminary. Therefore, future studies with larger samples, longitudinal designs, and taking into account the confounding effects of other critical variables (e.g., childhood trauma) should be implemented.

## 5. Conclusions

In conclusion, our results suggest that clinicians should carefully assess the presence of EMSs in individuals with FA symptoms in order to develop specific treatment approaches to address the management of addictive eating behaviors among obese and overweight patients with dysfunctional schemas. For example, the imagery re-scripting technique (i.e., accessing early experiences associated with EMSs development and direct schema modification) may be applied in order to manage loss of control over eating, which is a crucial symptom of FA. In a pilot study with bulimic patients, it has been reported that, compared to a control intervention, a single session of imagery intervention was associated with a significant decrease in depressive symptoms, as well as with a decreased urge to binge [51].

## Figures and Tables

**Table 1:** Descriptive statistics for the sample (*N* = 70).

Variables	Mean/*N*	SD/%
Age—mean (SD)	46.09	(9.95)
BMI—mean (SD)	29.65	(4.25)
School attainment ≤13 years—*N* (%)	9	(12.9)
Unemployed—*N* (%)	20	(28.6)
Married—*N* (%)	41	(58.6)
Tobacco use in the last 6 months—*N* (%)	9	(12.9)
Alcohol use in the last 6 months—*N* (%)	55	(78.6)
Menopause—*N* (%)	23	(32.9)
FA symptom count—mean (SD)	2.89	(1.71)
FA Diagnosis—*N* (%)	16	(22.9)
BES—mean (SD)	13.64	(9.51)
BES > 17 *N* (%)	20	(28.5)
HADS-A—mean (SD)	7.24	(4.47)
HADS-D—mean (SD)	6.34	(3.88)
Disconnection/Rejection—mean (SD)	126.49	(43.73)
Impaired Autonomy/Performance—mean (SD)	71.26	(24.28)
Impaired Limits—mean (SD)	46.83	(16.89)
Other-Directedness—mean (SD)	98.23	(28.61)
Over-vigilance/Inhibition—mean (SD)	103.99	(37.21)

Note: SD = standard deviation; *N* = number of cases; BMI = Body Mass Index; FA = Food Addiction; BES = Binge Eating Scale; HADS-A = Hospital Anxiety and Depression Scale-Anxiety subscale; HADS-D = Hospital Anxiety and Depression Scale-Depression subscale.

**Table 2:** Values of Pearson’s *r* correlation coefficient among variables in all samples (*N* = 70). Significant correlations are indicated by stars (*).

Variables	YFAS	BES	HADS-A	HADS-D	Disc/Rej	Imp Auton/Perfor	Impaired Limits	Other-Directedness	Overvig/Inhib	BMI	Age
YFAS	-										
BES	0.63 **	-									
HADS-A	0.31 **	0.21	-								
HADS-D	0.43 **	0.38 **	0.60 **	-							
Disc/Rej	0.60 **	0.41 **	0.38 **	0.50 **	-						
Imp Auton/Perfor	0.39 **	0.11	0.42 **	0.42 **	0.65 **	-					
Impaired Limits	0.46 **	0.26 *	0.21	0.32 **	0.58 **	0.62 **	-				
Other-Directedness	0.44 **	0.27 *	0.38 **	0.50 **	0.69 **	0.74 **	0.67 **	-			
Overvig/Inhib	0.42 **	0.21	0.37 **	0.45 **	0.59 **	0.66 **	0.81 **	0.81 **	-		
BMI	0.14	0.28 *	0.04	0.17	0.22	0.02	0.22	0.15	0.21	-	
Age	−0.11	0.01	0.12	0.33 **	−0.11	0.01	−0.17	−0.05	0.02	0.07	-

Note: - = 1.00 * *p* < 0.05; ** *p* < 0.01; YFAS = Yale Food Addiction Scale; BES = Binge Eating Scale; HADS-A = Hospital Anxiety and Depression Scale-Anxiety sub-scale; HADS-D = Hospital Anxiety and Depression Scale-Depression subscale; Disc/Rej = Disconnection/Rejection; Imp Auton/ Perfor = Impaired Autonomy/Performance; Overvig/Inhib = Over-vigilance/Inhibition; BMI = Body Mass Index.

**Table 3:** Results of the hierarchical linear regression analysis (*N* = 70).

Dependent Variable	Adjusted *R*^2^	*F*	Block	*R*^2^ Change	*F* Change	Independent Variables	Standardized Beta
YFAS total score	0.16	7.70 ^1^***	1	0.19 ***	7.01 ***	HADS-A	0.08
						HADS-D	0.38 **
							
	0.42	17.85 ^2^***	2	0.26 ***	31.21 ***	HADS-A	0.09
						HADS-D	0.17
						BES	0.55 ***
							
	0.50	9.76 ^3^***	3	0.11 *	3.12 **	HADS-A	0.04
						HADS-D	0.04
						BES	0.47 ***
						Disconnection/Rejection	0.29 *
						Impaired Autonomy/Performance	0.07
						Impaired Limits	0.14
						Other-Directedness	−0.07
						Over-vigilance/Inhibition	0.02

Note: * = *p* < 0.05; ** = *p* < 0.01; *** = *p* < 0.001; degrees of freedom: ^1^ 2:67, ^2^ 3:69, ^3^ 8:61; Abbreviations: YFAS = Yale Food Addiction Scale; HADS-A = Hospital Anxiety and Depression Scale-Anxiety subscale; HADS-D = Hospital Anxiety and Depression Scale-Depression subscale; BES = Binge eating scale.
